# Phenotypic analysis of peripheral B cell populations during *Mycobacterium tuberculosis* infection and disease

**DOI:** 10.1186/s12950-016-0133-4

**Published:** 2016-07-29

**Authors:** Willem J. du Plessis, Alana Keyser, Gerhard Walzl, André G. Loxton

**Affiliations:** 1 SA MRC Centre for TB Research, DST/NRF Centre of Excellence for Biomedical Tuberculosis Research, Division of Molecular Biology and Human Genetics, Faculty of Medicine and Health Sciences, Stellenbosch University, PO Box 241, Cape Town, 8000 South Africa; 2 Clinical Laboratory Sciences, Faculty of Health Sciences, University of Cape Town, Cape Town, South Africa

**Keywords:** B cells, Marginal zone, Plasma cells, Immuno-phenotyping, Biomarker, Immune activation

## Abstract

**Background:**

*Mycobacterium tuberculosis* (*Mtb*) remains an unresolved threat resulting in great annual loss of life. The role of B cells during the protective immunity to *Mtb* is still unclear. B cells have been described as effector cells in addition to their role as antibody producing cells during disease.

Here we aim to identify and characterize the frequency of peripheral B-cell subpopulations during active Tuberculosis and over treatment response. Analysis were done for both class switched (CS) and non-class switched (NCS) phenotypes.

**Methods:**

We recruited participants with active untreated pulmonary Tuberculosis, other lung diseases and healthy community controls. All groups were followed up for one week from recruitment and the TB cases till the end of treatment (month 6).

**Results:**

Peripheral blood samples were collected, stained with monoclonal antibodies to CD19^+^ cells, Immunoglobulin (Ig) M, plasma cells (CD 138^+^), marker of memory (CD27^+^), immune activation (CD23^+^) and acquired on a flow cytometer. Circulating Marginal zone B cells (CD19^+^IgM^+^CD23^−^CD27^+^) and memory phenotypes are able to distinguish between TB diagnosis and end of treatment. The frequency of mature B cells from TB cases are lower than that of other-lung diseases at diagnosis. A subpopulation of activated memory B cells (CD19^+^IgM^+^CD23^+^CD27^+^) cells are present at the end of TB treatment.

**Conclusions:**

This study identified distinctive B cell subpopulations present during active TB disease and other lung disease conditions. These cell populations warrants further examination in larger studies as it may be informative as cell markers or as effectors/regulators in TB disease or TB treatment response.

## Background

Tuberculosis (TB), remains an unresolved threat that is responsible for great mortality and morbidity in humans. Its causative agent, *Mycobacterium tuberculosis* (*Mtb*), was ultimately responsible for 9 million newly reported cases and 1.5 million deaths during 2013 [1]. Although great progress has been made on T cell based tuberculosis research, it is imperative that new avenues have to be explored and that previously underappreciated cell types are re-evaluated for their roles during the tuberculosis infection with the expectation of bringing an end to the epidemic. It is commonly accepted that B cell and antibody-mediated responses confers protection against extracellular pathogens and that the regulation and control of intracellular organisms are through cellular immune mechanisms.

There is increasing evidence that demonstrate B cells functioning as mediators (in both effector and regulatory roles) of immunity outside of their classically designated profession as the facilitators of humoral immunity. B cell activation by Toll-like receptor (TLR) antigens or whole organisms (like BCG or *Mtb*) can lead to a range of outcomes to the host, either by producing antibody, secreting cytokines (including interleukin (IL)-6, IL-10, and interferon (IFN)-gamma) or presenting antigen to naïve T cells [2–5]. B cell responses are beneficial to the host during infections and damaging during autoimmune disease. Conversely, B cells have the capacity to limit the hosts defence (inflammatory response) against pathogens and shield against autoimmune pathologies. This demonstrates that B cells can have distinct roles as drivers and regulators of immunity depending on the functional properties they gain following receptor activation and differentiation.

Although ongoing studies and literature supports the functional role of B cells during TB [6], the respective change in the frequency of the circulating B cell repertoire during active *Mtb* infection remains a topic for discussion as some studies report either a significant decrease [7] or increase [8] of peripheral blood B cell populations in actively infected patients.

Immuno-phenotyping has proven to be a very useful tool in the identification, monitoring and management of various clinical diseases [9–11]. Although recent publications have sought to develop in depth multicolour flow cytometric panels for the accurate delineation of various lymphocyte populations and subpopulations (including B cells) during immunodeficiencies [12, 13], very few studies exist that specifically assess immune-phenotypic change during active *Mycobacterium tuberculosis* infection [7, 14]. Little is known about the immune-phenotypic change of the B cell lineage during active *Mtb* as current literature largely focuses on the general B cell presence (primarily looking at CD19^+^ B cells only) [7, 14], rather than on an in-depth analysis of various populations and subpopulations. This results in a lack of knowledge pertaining to changes in B cell populations implicated in effector roles such as circulating memory B cells or plasma populations. It also does not elucidate the current activation state of B cells nor the expression of surface molecules, thus highlighting the need for further investigation regarding this matter.

In this brief preliminary report, a total of 96 participant samples spanning three groups (tuberculosis – active infection; 52 samples, other-lung disease; 24 samples, and healthy community controls; 20 samples) and various time points relating to treatment were used to assess the B cell repertoire in detail with the hope of identifying unique phenotypic differences between the groups that could suffice as biomarkers of disease. The primary contribution of this data would be to map the phenotypic distribution of B cells between these groups with a vast range, as it would include phenotypes for both IgM^+^ and IgM^−^ B cells. The actual isotype linked to the IgM^−^ phenotypes have not been determined for this study.

## Methods

### Patients

This study was done in the Western Cape Province of South Africa where a 2003 report showed that the TB detection rate was 678/100,000 population [15]. Previous publications have defined the characteristics associated with the economically depressed and disadvantaged metropolitan population of homogeneous ethnicity primarily found in the Western Cape Province, presenting high incidence rates of *Mtb* and transmission [16, 17]. The study participants included all attendees of the Infectious Diseases Clinics at Tygerberg Hospital, all community members of surrounding areas including Ravensmead, Uitsig, Adriaanse, Elsiesriver and local health care clinics. A total of 96 HIV negative participants, spanning three groups, were recruited for this study. All the study participants were also negative for Hep B. Fifty-two had active drug susceptible tuberculosis disease (on standard TB treatment regime) (TB disease status was confirmed by two separate positive sputum smear tests and a PCR for DNA of bacteria of the *Mtb* complex, by utilising the GeneXpert platform), 20 were healthy community controls (*Mtb* culture negative, Quantiferon test positive, therefor assuming latently infected with *Mtb*) and the third group consisted of 24 other-lung disease (OLD) patients. These OLD patients were all TB and HIV negative and presented with at least one of the following: a) febrile illness with chest symptoms, b) radiographic evidence of viral or bacterial pneumonia, c) bronchiectasis with acute exacerbation, or d) acute exacerbation of asthma or COPD (chronic obstructive pulmonary disease). The OLD patients were not on any steroid treatment at the time on recruitment. Table 1 summarizes the demographic data of study participants.

**Table 1:** Clinical and demographic data of the study participants

Group	Age Range	Gender-ratio (Male: Female)	BMI	HIV (+)	Other-lung Disease Diagnosis^a^	TB Type
Tuberculosis (*n* = 52)	18–50	3 M : 1 F	17.8 ± 1.7	0	n/a	Pulmonary
HC (*n* = 20)	19–50	4 M : 1 F	22.9 ± 6.3	0	n/a	n/a
OLD (*n* = 24)	25–64	1 M : 1 F	27.4 ± 8.2	0	Pneumonia (*n* = 10)	n/a
					Asthma (*n* = 2)	
					COPD (*n =* 1)	
					Pleural Effusion, Reactive (*n* = 1)	

OLD other-lung disease, HC healthy community control, BMI body mass index, COPD chronic obstruction of airways disease. ^a^All cases had to include symptoms of fever, coughing and other symptoms that would classify the case as acute and of exacerbated nature. Patients on systemic steroid therapy was excluded from the study

### B cell phenotyping

**Fig. 1:**
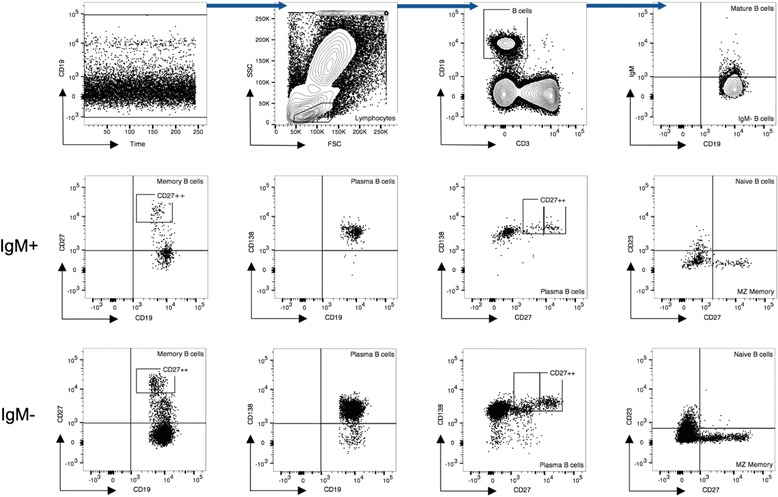
Flow cytometric gating strategy to identify the different peripheral B cell phenotypes. Populations for both IgM+ and IgM- are shown. MZ = marginal zone

Peripheral blood samples from the two control groups and patients were collected on various scheduled visits which included diagnoses, day 7 on treatment and at week 24 (end of TB treatment). White blood cells were obtained by subjecting each sample to a red blood cell lysing step using BD FACSlyse solution (BD Bioscience Pharmingen – San Jose, CA, USA). Leukocytes were stained following a standard procedure with anti-human CD3 (APC/Cy7, clone HIT3a), anti-human CD4 (PerCP/Cy5.5, clone OKT4), anti-human CD8a (Brilliant Violet 510, clone RPA-T8), anti-human CD19 (PE/Cy7, clone HIB19), anti-human CD23 (FITC, clone EBVCS-5), anti-human CD27 (PE, clone M-T271), anti-human CD138 (APC, clone DL-101) and anti-human IgM (Brilliant Violet, clone MHM-88). All antibodies were purchased from BioLegend (San Diego, California, United States of America). A total of 100,000 lymphocytes/sample were acquired on a FACS Canto II (BD Biosciences). All post acquisition analysis was done with FlowJo Software v10 (Tree Star Inc.) and the frequencies of the parent populations determined (Fig. 1). The assessed phenotypes (Fig. 1 showing the gating strategy using an treated TB case) were defined as follows: (1) Mature B cells (CD19^+^IgM^+^), (2) Activated B cells (CD19^+^IgM^+^CD23^+^), (3) Naïve B cells (CD19^+^IgM^+^CD23^+^CD27^+^), (4) Circulating Marginal Zone (MZ) B cells (CD19^+^IgM^+^CD23^−^CD27^+^), (5) Memory B cells (CD19^+^IgM^+^CD27^+^), (6) Plasmablasts with memory phenotype B cells (CD19^+^IgM^+^CD27^++^) (7) Total plasma cells (CD19^+^IgM^+^CD138^+^), (8) Plasmablasts cells (CD19^+^IgM^+^CD27^+^CD138^+^) and (9) Plasmablasts with memory phenotype B cells (CD19^+^IgM^+^CD138^+^CD27^++^). All IgM^+^ phenotypes are referred to as non-class switched (NCS) and IgM^−^ phenotypes as class switched (CS) in text.

### Statistical analysis

Differences in the frequency of B cell subsets between the groups were analysed using the non-parametric analysis with a Mann-Whitney correction and performed by Dr Justin Harvey (Stellenbosch University). All analysis were performed with the Statistica 12 software (Statsoft, Ohio, USA).

## Results and discussion

### Circulating marginal zone B cells and memory phenotypes distinguish between TB diagnosis and end of treatment

**Fig. 2:**
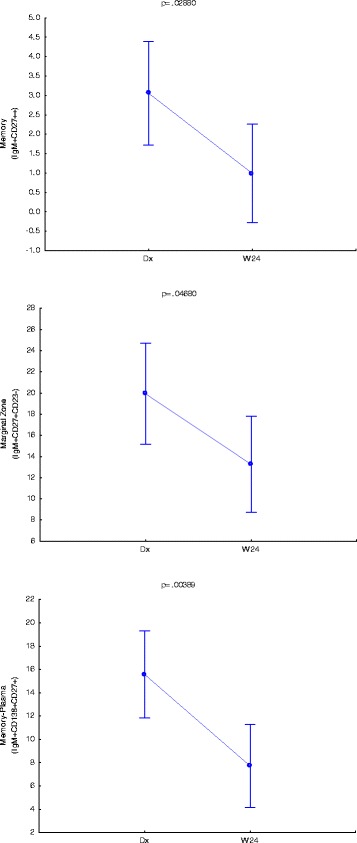
IgM+ B cell phenotypes that are significantly different at TB diagnosis and end of treatment (W24). Vertical bars denote 95 % confidence. Dx = diagnosis, W24 = week 24

**Fig. 3:**
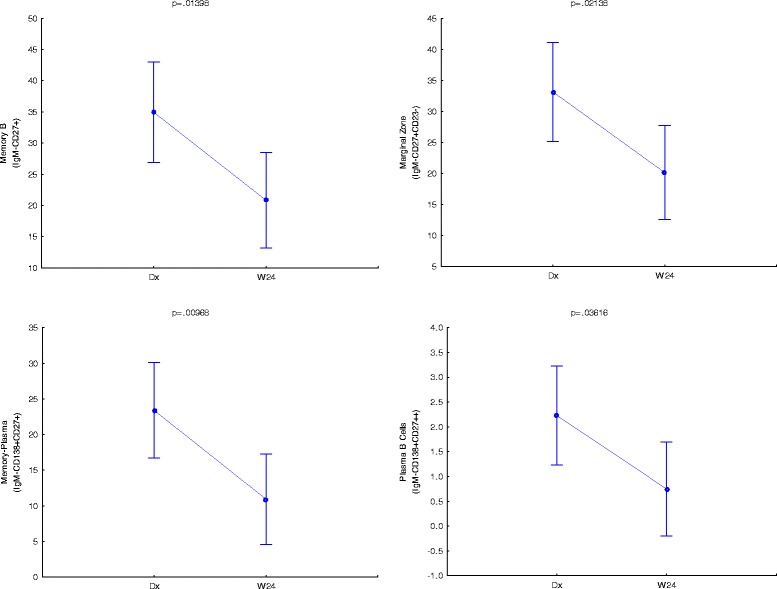
IgM- B cell phenotypes that are significantly different between TB diagnosis and end of treatment (W24). Vertical bars donate 95 % confidence. Dx = diagnosis, W24 = week 24

**Fig. 4:**
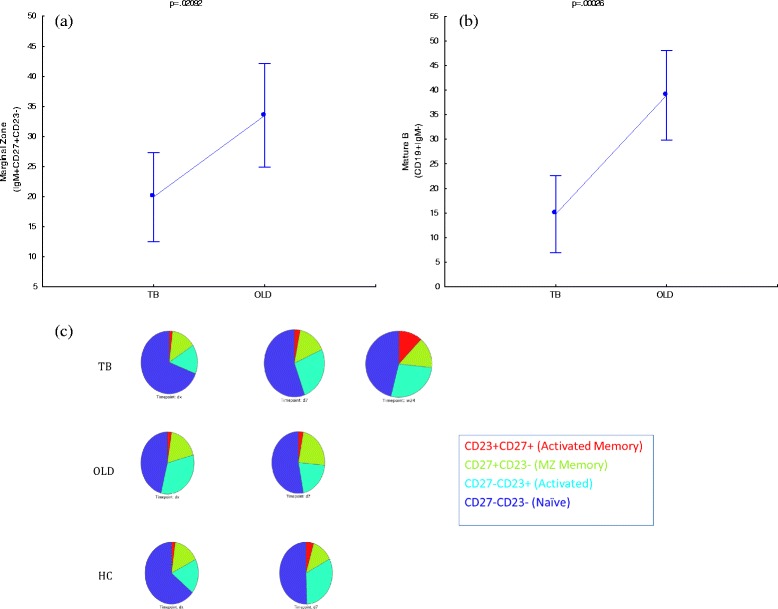
**a** IgM+ Marginal Zone B cells and **b** IgM- Mature B cells are significantly different between TB and OLD at diagnosis. TB = tuberculosis, OLD = other-lung disease. **c** Frequency distribution of circulating Marginal Zone B cells between groups over time. Dx = diagnosis, d7 = day 7 and w24 = week 24

We firstly aimed to identify phenotypes that were significantly different at diagnosis and the end of treatment (week 24). In Fig. 2 it is seen that the CD27^high^ memory B cells (CD19^+^IgM^+^CD27^++^), *p* = 0.02880, and plasmablasts B cells (CD19^+^IgM^+^CD138^+^CD27^+^), *p* = 0.00389, populations were significantly higher at diagnosis as when compared to levels at the end of treatment. Sebina et al. [18] observed that UK donors who previously lived in, or visited areas denoted as highly TB-endemic had higher frequencies of memory B cells in their peripheral blood as compared to their counterparts who have not. This study also reported that BCG elicited the production of long-lived mycobacteria-specific memory B cells [18], which supports the notion of high observed frequencies of memory B cells at diagnosis as BCG vaccination is a common practice at birth in South Africa. It has been shown that the maintenance of memory B cells are dependent on the presence of antigen, and that they are lost within 10–12 weeks following its absence [19]. Although this is opposite to other publications stating the longevity of B cells [18, 20–22], it corresponds to the observed result of lower memory B cell frequencies at the end of treatment. The high numbers of TB-specific (or BCG-specific, or mycobacteria-specific) memory B cells might be maintained in this population due to the continual exposure to antigen (either *M.tb* exposure in this high prevalence setting, or environmental or non-tuberculous mycobacterial exposure). It is reasonable that the decrease of memory B cells towards the end of treatment don’t relate to a complete loss of memory B cells, but rather represents an overall decrease in line with the reduction of bacterial burden. Figure 3 shows that memory based B cell phenotypes were significant in the CS cohort as well with memory B cells (CD19^+^IgM^−^CD27^+^); *p =* 0.01398, plasmablasts B cells (CD19^+^IgM^−^CD138^+^CD27^+^); *p =* 0.00968 and plasmablasts with memory phenotype a (CD19^+^IgM^−^CD138^+^CD27^++^); *p =* 0.03616. This continual significance in both NCS and CS phenotypes strengthens their importance as distinguishing factors. Circulating Marginal zone B cells (CD19^+^CD27^+^CD23^−^) were also significant in both NCS (*p =* 0.04680) and CS (*p =* 0.02138) analyses and between groups over time (Fig. 4). Together these results warrants further research into these phenotypes as potential biomarkers for treatment response.

### Circulating marginal zone- and Mature B cells can distinguish TB from other-lung diseases at diagnosis

In the attempt to identify phenotypes that were unique to tuberculosis when compared to other-lung based diseases, two results showed to be significant. The first was NCS marginal zone (MZ) B cells (CD19^+^IgM^+^CD27^+^CD23^−^) with *p =* 0.02092 and secondly CS mature B cells were significant with *p =* 0.00026 (Fig. 4). With both of these phenotypes significantly lower in peripheral circulation during active TB disease (especially the CS mature B cells), it raises the question whether TB actively suppresses the B cell repertoire during disease. The NCS mature B cell repertoire does not recover to baseline levels during the first week of treatment as one would expect (in line with chemotherapeutic treatment alleviating bacterial burden). These findings support the notion that there is a possible underlying mechanism exploited by *Mtb* that could be crucial to the management of the infection as both MZ and mature B cells are implicated in effector functions of the adaptive immune system, as seen with the overexpression of programmed death 1 (PD-1) on lymphocyte frequencies during active TB infection [23].

### Class switched and non-class switched mature B cells distinguish between tuberculosis, other-lung based diseases and healthy controls

**Fig. 5:**
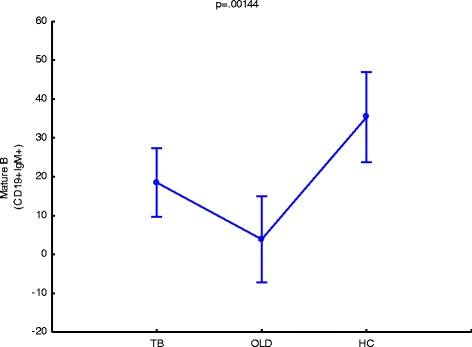
IgM+ Mature B cells are significantly different at diagnosis when compared to other disease states. *P*-Values represent significant difference, where an alpha level of 95 % is used. TB = tuberculosis, OLD = other-lung disease, HC = healthy control

**Fig. 6:**
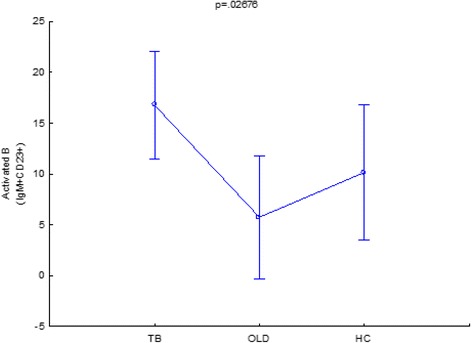
IgM+ Activated B cells are significantly different at diagnosis when compared to other disease states. An alpha level of 95 % was used in the LSD test, whose results is displayed in the accompanying table. *P*- Values show significance between specific comparisons. TB = tuberculosis, OLD = other-lung disease, HC = healthy control

Class switched mature B cells from participants with active TB are present in significantly different levels when compared to other-lung diseases (*p =* 0.043711) and the healthy control group (*p =* 0.024488) (Fig. 5). Mature B cells were not only able to distinguish between these groups in the class switched category, but also in the non-class switched category where the difference between TB and OLD was highly significant (*p =* 0.000016) and between TB and the healthy control group where *p* = 0.025939 (not shown)). An interesting observation is that the class switched mature B cells from TB is present at higher frequencies when compared to the OLD group (Fig. 5), but that the inverse is observed with non-class switched mature B cells where they are present in significantly lower levels when compared to the OLD group (not shown). This would suggest that a strong preference is displayed towards class switched mature B cells during active tuberculosis. The differences present in the CS and NCS mature B cells are consistent when compared during the first week of treatment (not shown), where the NCS mature B cells are present in higher frequencies as when compared to the class switched mature B cells. Further in this study, NCS activated B cells in TB could distinguish from OLD (*p =* 0.008391), but not from healthy controls (*p =* 0.122877) (Fig. 6). This result identifies another combination of markers that could be used to distinguish TB from other lung based diseases and should be further investigated as a measure in the early diagnosis of TB.

## Conclusion

This pilot study identified unique variations in the B cell repertoire during active tuberculosis infection when compared to healthy controls, other-lung based diseases and over the course of TB treatment. The first observation of memory-based phenotypes being the major distinguishers between diagnosis and end of treatment in both class switched and non-class switched phenotypes holds promise as markers for treatment response. The second important finding of this study is that circulating marginal zone B cells could not only distinguish between TB diagnosis and the end of treatment, but also has significantly different frequencies when compared to other-lung based diseases making it a candidate as biomarker for not only treatment response, but distinguishing active TB disease from other-lung based diseases. The observation that NCS and CS mature B cells could best distinguish between TB and the two control groups (healthy controls and other lung diseases) at diagnosis, but that their respective peripheral frequencies are present at an inverse level. Taken together, these results show that mainly B cell phenotypes implicated in activation and subsequent effector functions are influenced by TB and warrants further research to confirm their potential as biomarkers for TB disease and treatment response. These results are further strengthened if the assumption holds that only 10 % of the ongoing immunologic response is represented in the peripheral blood when compared to the site of disease, namely the lungs. Further studies is thus needed to replicate this experiment on broncho-alveolar lavage (BAL) fluid from active TB diseased participants and other lung diseases to ascertain if the phenotype remains the same. Although the results from the BAL fluid experiment might give a better representation of the on-site B cell frequencies, the challenges associated with obtaining the bio fluid and characterisation of the (mostly activated) cells could hamper the process of finding biomarkers of disease or TB treatment response.

## Abbreviations

BAL, broncho alveolar lavage; COPD, chronic obstructive pulmonary disease; CS, class switched; IFN, interferon; M.tb, mycobacterium tuberculosis; NCS, non class switched; OLD, other lung diseases; PD-1, programmed death 1; TB, tuberculosis; TLR, toll like receptor
